# Description of *Laimaphelenchus sinensis* n. sp. (Nematoda: Aphelenchoididae) from declining Chinese pine, *Pinus tabuliformis* in Beijing, China

**DOI:** 10.21307/jofnem-2020-019

**Published:** 2020-03-17

**Authors:** Jianfeng Gu, Munawar Maria, Yiwu Fang, Lele Liu, Yong Bian, Xianfeng Chen

**Affiliations:** 1 Technical Centre of Ningbo Customs (Ningbo Inspection and Quarantine Science Technology Academy), Huikang No. 8, Ningbo 315100, Zhejiang, P.R. China; 2 Laboratory of Plant Nematology, Institute of Biotechnology, College of Agriculture & Biotechnology, Zhejiang University, Hangzhou 310058, Zhejiang, P.R. China; 3 Technical Centre of Beijing Customs, No. 6 Tianshuiyuan Street, Chaoyang District, Beijing 100026, P.R. China

**Keywords:** *Laimaphelenchus*, Chinese pine, n. sp., Molecular, Morphology, Insect association, Phylogeny, Taxonomy

## Abstract

*Laimaphelenchus sinensis* n. sp. isolated from declining Chinese pine, *Pinus tabuliformis* Carrière, is described and characterized morphologically and molecularly. The new species has four incisures in the lateral field and the excretory pore situated posterior to the nerve ring; the female has a vulval flap and vaginal sclerotization is quite prominent in majority of specimens. The female tail is conoid, ventrally curved having a single stalk-like terminus with 8 to 10 projections. The male spicules are 14.0 (13.2-15) μm long along curved median line and tail is ventrally curved typical of the genus; however, the projections are less prominent as compared to those of female. The male has two pairs of caudal papillae and Bursa is absent. Phylogenetically, the ribosomal DNA sequences of the new species placed it within *Laimaphelenchus* clade and are morphologically similar to *L. persicus*, *L. preissii*, *L. simlaensis* and *L. unituberculus*.

Genus *Laimaphelenchus* has been defined by the presence of pedunculate tubercles that expanded to finger-like projections on the tail terminus, although some species with this character have been demonstrated to be polyphyletic and transferred to Aphelenchoides (Zhao et al., 2006a, 2007; Asghari et al., 2012; Carta et al., 2016; Maleita et al., 2018). At present, the genus contains 15 species distributed across different climatic zones and environment (Hunt, 1993; Peneva and Chipev, 1999; Asghari et al., 2012; Fang et al., 2019). Members of this genus are known to exhibit a global distribution as they have been reported from six continents (Hunt, 1993; Swart, 1997; Peneva and Chipev, 1999; Zhao et al., 2007; Negi et al., 2009; Pedram et al., 2018). Due to the presence of potential pest species in family Aphelenchoididae, members of this family are diagnosed with caution. However, none of the *Laimaphelenchus* species were reported to cause potential damage to conifers (Raghavendra and Newcombe, 2013), although their possible association with oak decline syndrome was suggested by Pedram et al. (2018).

During the present study, a population of *Laimaphelenchus* species was isolated from declining Chinese pine, *Pinus tabuliformis* Carrière, in Beijing, China, in November, 2018. The population was examined carefully and preliminary studies reveal the status of this species as a new species. Therefore, the objectives of the study are: to provide morphological and molecular characterization of *L. sinensis* n. sp.; and to demonstrate phylogenetic relationships of the new species with related aphelenchids.

## Materials and methods

### Nematode isolation and morphological study

Several twigs collected from declining Chinese pine (*Pinus tabuliformis*) were sliced into small pieces approximately 1 cm wide. The nematodes were isolated by the modified Baermann funnel technique for 24 hr. Adults for observation and measurements were collected from the declining twig samples as the cultures were unsuccessful. Permanent slides were prepared by heat-killed nematodes fixed with FA 4:1 and ethanol-glycerin dehydration according to Seinhorst (1959) as modified by De Grisse (1969). Morphometrics, drawings and light micrographs of nematodes were made with the aid of a Zeiss microscope equipped with a Zeiss AxioCam MRm CCD camera (Carl Zeiss Shanghai Co. Ltd. Shanghai, China).

### Molecular and phylogenetic analyses

DNA samples were prepared according to Li et al. (2008). Four sets of primers (synthesized by Majorbio, Shanghai, China) were used in the PCR analyses to amplify the near full length 18 S, full length ITS region and D2-D3 expansion segments of the 28 S ribosomal RNA genes (rDNA). The near full length 18 S region was amplified as two partially overlapping fragments; for the first fragment, 988 F (5-CTC AAA GAT TAA GCC ATG C-3) and 1912R (5-TTT ACG GTC AGA ACT AGG G-3) were used and for the second fragment 1813F (5-CTG CGT GAG AGG TGA AAT-3) and 2646 R (5-GCT ACC TTG TTA CGA CTT TT-3) (Holterman et al., 2006). The full length ITS region was amplified with the forward primer TW81 (5’-GTT TCC GTA GGT GAA CCT GC-3’) and the reverse primer AB28 (5’-ATA TGC TTA AGT TCA GCG GGT-3’) (Joyce et al., 1994). The 28 S D2-D3 region was amplified with the forward primer D2A (5’-ACA AGT ACC GTG AGG GAA AGT TG-3’) and the reverse primer D3B (5’-TCG GAA GGA ACC AGC TAC TA-3’) (De Ley et al., 1999). PCR conditions were as described by Li et al. (2008) and Ye et al. (2007). PCR products were separated on 1.5% agarose gels and visualized by staining with ethidium bromide. PCR products of sufficiently high quality were sent for sequencing by Invitrogen, Shanghai, China.

The newly generated near full length 18 S and 28 S D2-D3 rDNA sequences of *L. sinensis* n. sp. were compared with other aphelenchid sequences available in GenBank using the BLAST homology search program (Altschul et al., 1990). The alignments of selected sequences were conducted with MAFFT (Katoh and Standley, 2013) with the default parameters and edited with AliView (Larsson, 2014). The best-fitted model of DNA evolution and the base frequency, the proportion of invariable sites and the gamma distribution shape parameters and substitution rates were obtained using jModelTest2 (Darriba et al., 2012) with the Akaike information criterion. The phylogenetic tree for each gene was obtained separately using MrBayes 3.2.3 (Ronquist and Huelsenbeck, 2003) with four chains (three heated and one cold). The number of generations for the total analysis was set to 1 × 10^7^, with the chain sampled every 1,000 generations and the burn-in value set at 25%. The Markov chain Monte Carlo method within a Bayesian framework was used to estimate the posterior probabilities of the phylogenetic trees using the 50% majority rule (Larget and Simon, 1999). The consensus trees were selected to represent the phylogenetic relationships as well as the branch length and support level, all visualized using TreeGraph 2 (Stöver and Müller, 2010).

## Results

### Systematics

*Laimaphelenchus sinensis* n. sp.

(Figs 1 and 2).

**Figure 1:**
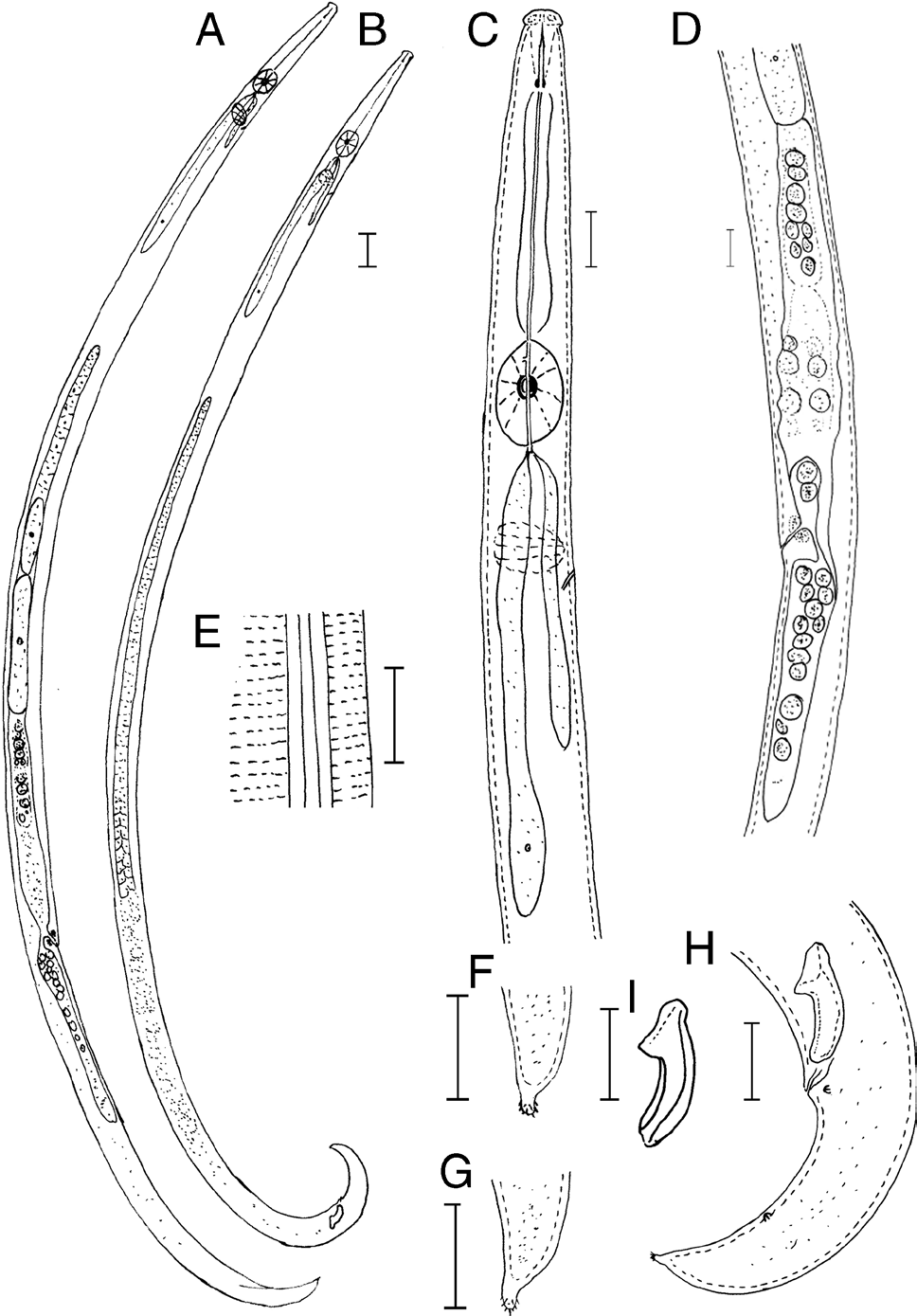
Line drawings of *Laimaphelenchus sinensis* n. sp. A: Entire female; B: Entire male; C: Anterior region; D: Female posterior region showing vulva and post-uterine sac; E: Lateral lines F, G: Female tail terminus; H: Male tail; I: Spicule. (Scale bars = A, B = 20 μm; C-I = 10 μm).

**Figure 2:**
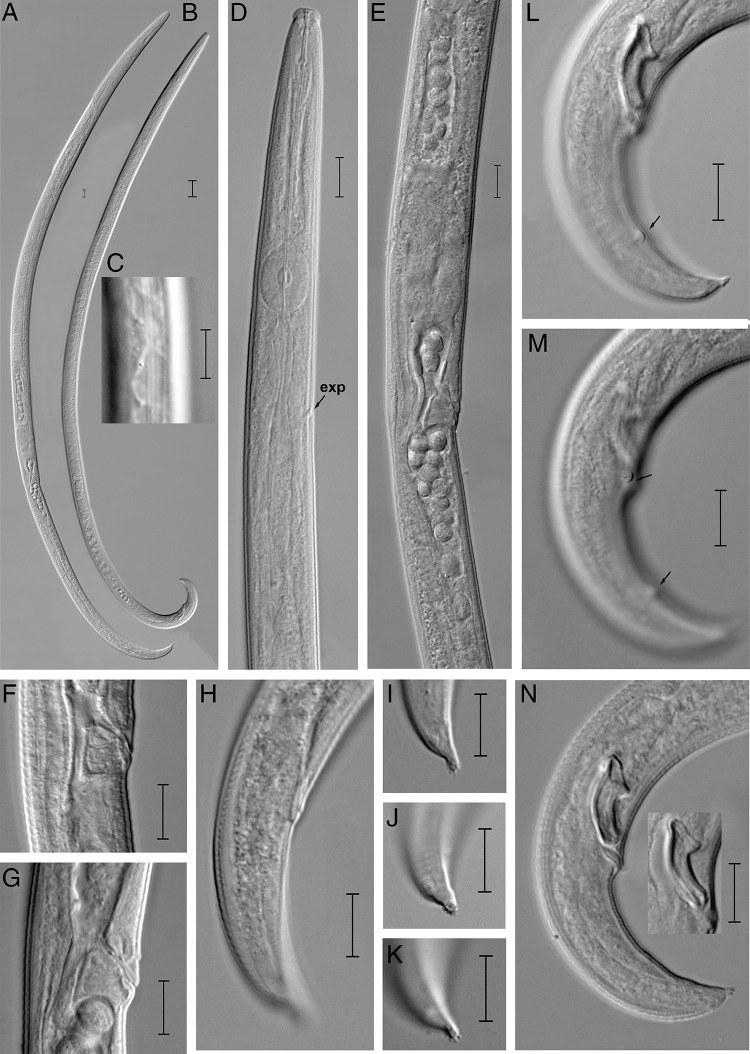
Light photomicrographs of *Laimaphelenchus sinensis* n. sp. A: Entire female; B: Entire male; C: Lateral lines; D: Anterior region; E: Female posterior region showing vulva and post-uterine sac; F, G: Vulval regions; H: Female tail; I-K: Female tail terminus; L-N: Male tails arrows showing position of caudal papillae (Scale bars = A, B = 20 μm; C-N = 10 μm; Abbreviations: ex, excretory pore).

#### Measurements

Measurements of the new species are given in Table 1.

**Table 1.** Morphometrics data for *Laimaphelenchus sinensis* n. sp.

	Female		Male
Character	Holotype	Paratypes	Paratypes
n	–	6	5
L	914	968±46.1 (914-1064)	876±69.1 (750-956)
a	42.1	41.5±1.9 (38.6-44.8)	46.2±2.6 (42.4-50)
b	11.2	11.7±0.6 (11.2-12.7)	10.5±0.6 (9.4-11.1)
b’	4.3	4.8±0.4 (4.3-5.6)	4.6±0.3 (4.1-4.9)
c	25.3	28±3.1 (25-32.9)	19.9±1.4 (17.9-22.3)
c’	2.6	2.6±0.3 (2.1-2.9)	2.7±0.2 (2.4-2.9)
V or T	70.5	69.1±0.9 (67.7-70.7)	70.8±4.4 (63.4-76.5)
Lip region height	2.3	2.4±0.1 (2.2-2.6)	2.7±0.1 (2.6-2.8)
Lip region width	6.9	7.1±0.3 (6.8-7.4)	7.1±0.6 (6.4-7.9)
Stylet length	12.2	12.3±0.3 (11.8-12.6)	12.3±0.6 (11.1-12.9)
Body diam.	21.7	23.4±1.7 (21.6-26.4)	19±1.1 (17.7-20.9)
Median bulb width	18.9	13.2±0.7 (12.4-14)	12±0.7 (11-12.8)
Median bulb length	12.6	18.5±0.5 (17.6-18.9)	17.4±0.8 (16.2-18)
Median bulb length/diam. ratio	1.5	1.4±0.1 (1.3-1.5)	1.5±0.1 (1.4-1.5)
Excretory pore from anterior end	92.2	96.3±4.7 (88-104)	83.2±2.5 (80.1-87)
Ovary length or testis	430	454±24 (420-480)	622.6±76.6 (476-690)
Post-uterine sac	121	130.3±6.1 (119-138)	–
Vulva to anus distance	270	264.2±20.9 (233-300)	–
Post-uterine sac length/vulva to anus (%)	44.8	49.8±5.5 (39.7-55.8)	–
Anal (cloacal) body diameter	14.1	13.3±0.7 (12-14.1)	16.3±1.0 (15.3-18.2)
Tail length	36.1	34.9±3.2 (29-38.6)	44.1±1.8 (41.8-46.4)
Spicule (curved median line)	–	–	14.0±0.6 (13.2-15)
Spicule (Chord)	–	–	15.6±0.9 (14.1-16.6)

**Note:** All measurements are in μm and in the form of mean ± SD (range)

### Description

#### Female

Body is slender, cylindrical, and J-shaped when heat killed. Cuticle has fine annulations. Lateral field has four incisures. Lip region is convex in lateral view, offset, more than twice as broad as high. Stylet is 12.3 (11.8-12.6) μm long, divided into two parts with small basal swellings and conus occupying *ca* 40% of its total length. Procorpus is cylindrical, and metacorpus (median bulb) is strongly developed and oval shaped, with centrally situated valves. Dorsal pharyngeal gland orifice opens into lumen of metacorpus with *ca* one metacorpal valve length anterior to metacorpal valve. Pharyngo-intestinal junction is one metacorpal valve length posterior to the base of metacorpus. Nerve ring is less than one body diam. length posterior to metacorpus. Pharyngeal gland lobe is slender, overlapping intestine dorsally. Excretory pore is located slightly posterior to nerve ring. Reproductive tract is mono-prodelphic and located to the right of intestine. Organs are arranged as ovary, oviduct, spermatheca, crustaformeria, uterus, vagina + vulva and post-uterine sac. Single, outstretched ovary, developing oocytes arranged in 1 to 2 rows, and several well-developed oocytes arranged in single row. Oviduct is short and connected with an ovoid- to oblonged-shaped spermatheca, filled with sperm cells. Spermatheca is formed by thick tissue, forming an expansion in the gonad, i.e. not forming a clear branch. Crustaformeria is not conspicuous. Vagina is slightly inclined anteriorly to body axis, with massive sclerotization, but sometimes invisible. Vulva is a traverse slit, and anterior vulval lip is modified into a small vulval flap covering the vulval region. Post-uterine sac and vagina are usually closed with no special structure such as a pair of three-celled structures. Post-uterine sac is long, extending for *ca* 39.7 to 55.8% of vulval–anus distance, and sometimes filled with sperm. Anus is distinct, and area at anal lips is slightly elevated. Tail is conoid, slightly ventrally curved with a mucron of about 2 μm long, and appears like a stalk-like terminus with multiple (8-10) projections.

#### Male

Body is slender, cylindrical, and slightly ventrally arcuate when heat-relaxed. Cuticle and anterior body region are similar to female. Testis, outstretched, located on the left side of intestine. Anterior part of testis contains developing spermatocytes in a single row and gradually develops into two rows. Cloacal lips are non-protruded. Spicules are paired, condylus is broad squarish to rounded shaped with triangular rostrum, capitulum is straight to slightly bent in some individuals, and lamina/calomus is complex and smoothly curved to distal end. Distal ends of spicule have rounded terminus. Two pairs of papillae are present: one pair is subventral precloacal papillae (P2) located at the same level of cloacal opening, and the other pair is subventral postcloacal papillae (P3) located at mid of the tail. Tail is conoid ventrally curved with several tubercles. Bursa is absent.

#### Type host and locality

The type material was isolated from declining Chinese pine, *Pinus tabuliformis* Carrière, in Beijing, China, on November, 2018.

#### Type specimens

Holotype female, four male and four female paratypes (slide numbers BJ1-1 to BJ1-3) were deposited in the nematode collection of Ningbo Entry-Exit Inspection and Quarantine Bureau, China. One paratype male and two paratype females (slide numbers T551) were deposited in the Canadian National Collection of Nematodes, Ottawa, ON, Canada.

#### Etymology

The species epithet is formed from the country of origin.

#### Differential diagnosis

The *L. sinensis* n. sp. can be characterized by the lateral field with four lines and the excretory pore situated posterior to the nerve ring. The male spicules are 14.0 (13.2–15) μm long along the curved median line, condylus is broad squarish to rounded shaped with triangular rostrum, capitulum is straight to slightly bent in some individuals, and distal ends of spicule have a rounded terminus. Two pairs caudal papillae are present. Bursa is absent. Female have a vulval flap. Tail is conoid and ventrally curved with a single stalk-like terminus with 8 to 10 projections.

The new species has a vulval flap and four lateral lines, whereas none of the other *Laimaphelenchus* species exhibits this combination except these four species: *L. persicus* (Asghari et al., 2012); *L. preissii* (Zhao et al., 2006b); *L. simlaensis* (Negi et al., 2009) and *L. unituberculus* (Bajaj and Walia, 2000).

The new species can be differentiated from *L. persicus* by tail terminus morphology (single stalk with 8 to 10 projections vs 4 pedunculate tubercles ending with 4 to 6 finger-like protrusions), longer female body L = 968 (914–1064) vs 763 (615–925) μm, higher c value = 28 (25–32.9) vs 21.8 (17.5–24.7) and smaller spicule lengths 14.0 (13.2–15) vs 20.4 (19–21.0) μm; from *L. preissii* by size of anterior vulval lip (smaller vs elongated, well developed), spicule morphology (condylus broad squarish to rounded shaped with triangular rostrum distal ends of spicule with rounded terminus vs condylus and rostrum broad ellipsoidal with bluntly rounded terminus), bursa (absent vs present), smaller spicule lengths 14.0 (13.2–15) vs 22 to 28 μm, shorter body length of male 876 (750–956) vs 1,088 (1,000–1,218) and female 968 (914–1,064) vs 1,185 (1,007–1,386) μm and smaller of female tail 34.9 (29–38.6) vs 44 (32–64) μm; from *L. simlaensis* by the spicule morphology (condylus broad squarish to rounded shaped with triangular rostrum vs condylus broad rounded with pointed rostrum) and length 14.0 (13.2–15) vs 16 to 18 μm, spicule devoid of gubernaculm like structure vs present, female tail terminus morphology (single stalk with 8 to 10 projections vs 3 to 5 finger-like fine processes and male having 2 pairs of caudal papillae vs 3; from *L. unituberculus* by posterior position of excretory pore from anterior end (posterior to nerve ring vs at the same level of it), vaginal scelorotization (medium vs massive), the spicule morphology (condylus broad squarish to rounded shaped with triangular rostrum vs condylus conoid with pointed rostrum and straight condylus) longer body length of male 876 (750–956) vs 640 (520–720) μm and female 968 (914–1,064) vs 740 (690–800) μm and male having 2 pairs of caudal papillae vs 3.

#### Molecular profiles and phylogenetic status

The new species was molecularly characterized using near full length 18 S, full length of ITS region and D2-D3 expansion segments of 28 S rDNA sequences which were deposited in GenBank under the following accession numbers: MN401302 (18 S, 1,675 bp), MN 401304 (ITS, 820 bp) and MN401303 (28 S, 726 bp). The 18 S data set had 1,874 characters and the 28 S data set had 976 characters after alignment and post-editing. Phylogenetic relationships among the isolates for each data set were assessed using Bayesian inference, with *Aphelenchus avenae* Bastian, 1,865 (JQ348399) for 18 S and (JQ348340) for 28 S as the outgroup taxon. The 50% majority rule consensus phylogenetic trees were generated from both datasets under the TrN + I + G and TIM2 + I + G models, respectively. Previously, none of the *Laimaphelenchus* species was characterized using ITS sequence; hence, ITS tree was not constructed during this study.

**Figure 3:**
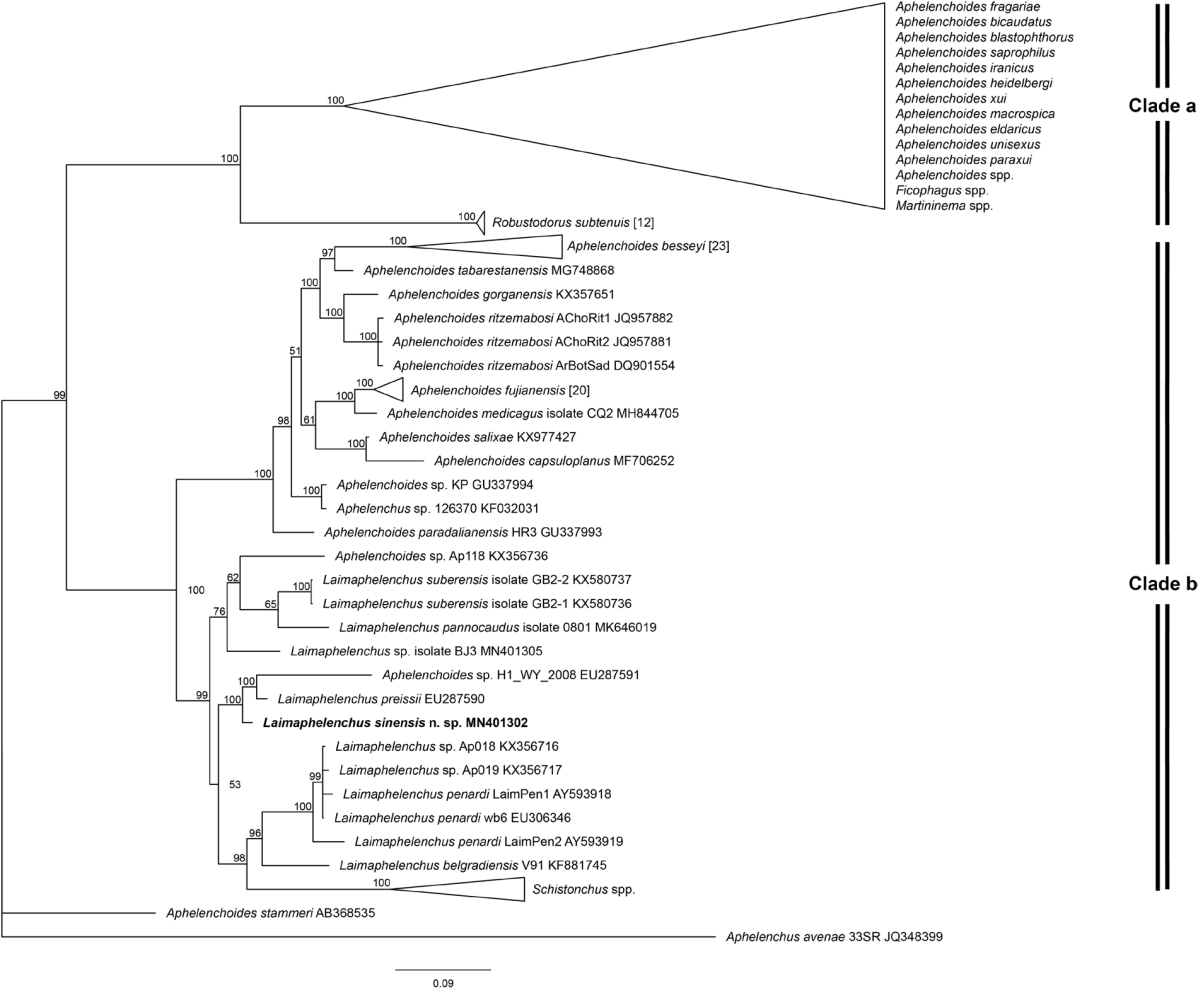
Phylogenetic relationships of *Laimaphelenchus sinensis* n. sp. and aphelenchid nematodes based on full length of 18 S rDNA. The 100001st Bayesian tree inferred from 18 S rDNA under TVM + I + G model. *Aphelenchus avenae* (JQ348399) served as the outgroup species. Posterior probability values exceeding 50% are given on appropriate clades.

The 18 S phylogenetic tree (Fig. 3) demonstrated *L. sinensis* n. sp. clustered with *L*. *preissii* and an unidentified *Aphelenchoides* sp. (EU287591) as an independent clade with high support value (posterior probability = 100). The sequence comparison of the new species with *L*. *preissii* showed a sequence divergence of 1.67% (28/1,678 bp). Furthermore, the 18 S sequence divergence of the new species with other molecularly characterized *Laimaphelenchus* species having a vulval flap, i.e. *L. belgradiensis* (KF881745) and *L. penardi* (EU306346, AY593918, AY593919) ranged from 4.88 to 6.68%.

**Figure 4:**
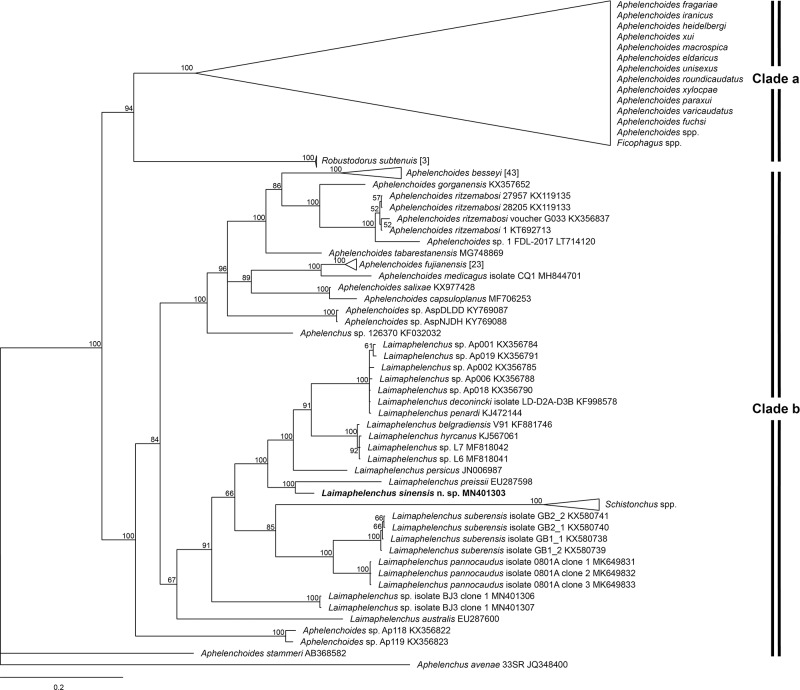
Phylogenetic relationships of *Laimaphelenchus sinensis* n. sp. and aphelenchid nematodes based on D2-D3 expansion segments of 28 S rDNA. The 100001st Bayesian tree inferred from 28 S rDNA under TIM2 + I + G model. *Aphelenchus avenae* (JQ348400) served as the outgroup species. Posterior probability values exceeding 50% are given on appropriate clades.

The phylogenetic tree of 28 S D2-D3 (Fig. 4) is similar to the 18 S tree and *L. sinensis* n. sp. appears as a sister taxon to *L. preissii*. The sequence divergence of these two species is 12.67% (91/718 bp). Furthermore, the 28 S sequence divergence of the new species with other molecularly characterized *Laimaphelenchus* species having a vulval flap, i.e. *L. belgradiensis* (KF881746), *L. deconincki* (KF998578), *L. hyrcanus* (KJ567061) and *L. persicus* (JN006987)) ranged from 12.72 to 13.43%.

## Discussion

Only the *Laimaphelenchus* species from the United States (*L. pensorobins*, *L. penardi*, *L. pannocaudus* and *L. phaseolini*) were found in association with insects. The rest of the species were either described from the dead/weakened branches of coniferous/deciduous trees or from the soil rhizosphere and presumed to be associated with mosses or lichens growing on the host trees (Massey, 1966, 1974; Bajaj and Walia, 2000; Zhao et al., 2006a, 2007; Negi et al., 2009; Asghari et al., 2012; Oro, 2015; Maleita et al., 2018). Regarding this, it can be presumed that the *Laimaphelenchus* species may utilize insect vectors for their dispersal if there is any biological state associated with insects yet to be discovered.

The discovery of *L. helicosoma* from the remote region of Antarctica (Maslen, 1979; Peneva and Chipev, 1999) and the detection of *L. penardi*, *L. deconicki* (Asghari and Eskandari, 2014; Azizi et al., 2015) from Iran indicate the ancient origin of genus *Laimaphelenchus* which might date back to the geographical period even prior to the breakup of Pangaea and later it might have spread during the historic land connections. However, *Laimaphelenchus* species are not regarded as quarantine pests; therefore, the biogeographical distribution of this genus is poorly known.

The new species is the first *Laimaphelenchus* species described from China. It was discovered during the routine nematode inventory survey and no insects were detected on the wood samples. Additionally, this is the first *Laimaphelenchus* species that has been recovered from *Pinus tabuliformis*. The details regarding species biology and insect associations are unknown. However, this gap in the knowledge is a framework for further studies and will hopefully stimulate future research.
